# The chain mediating role of social support and positive coping between neuroticism and depressive symptoms among graduate students

**DOI:** 10.3389/fpsyt.2024.1424983

**Published:** 2024-09-25

**Authors:** Peng Wan, Jinsheng Hu, Qingshuo Yang

**Affiliations:** Department of Psychology, Liaoning Normal University, Dalian, China

**Keywords:** graduate students, neuroticism, social support, positive coping, depressive symptoms

## Abstract

**Introduction:**

Graduate students face unprecedented levels of neuroticism and pressure compared to their peers. Despite existing research examining the connection between neuroticism and depressive symptoms, a gap in the current understanding of the mediating mechanisms that act on this relationship, especially among this specific student population.

**Methods:**

This study investigated the potential chain-mediating roles of social support and positive coping in the relationship between neuroticism and depressive symptoms among graduate students. The participants were 1845 graduate students who provided demographic information and complete assessments including the Eysenck Personality Questionnaire (EPQ), Depression Rating Scale (BDI-II), Social Support Rating Scale (SSRS), and Coping Style Questionnaire (SCSQ). The analysis involved correlational analysis and a chain mediation model was used to investigate the associations among neuroticism, social support, positive coping mechanisms, and depressive symptoms.

**Results:**

The results show significant correlations among neuroticism, depressive symptoms, social support, and positive coping. Moreover, our findings verify that neuroticism affects depressive symptoms in graduate students through three pathways: the mediating effect of social support, the mediating effect of positive coping, and the chain-mediating effect of social support and positive coping.

**Discussion:**

Overall, the hypothesized chain model conclusively fits the data: Neuroticism directly affects depressive symptoms among graduate students and influences depressive symptoms through the mediating effects of social support and positive coping, as well as the chain mediating effects of these two variables.

## 1 Introduction

Depressive symptoms have long been a major global concern (1, 2). Feelings of detachment and chronically low mood are hallmarks of depression. Key symptoms include feelings of worthlessness, helplessness, and despair along with various physical and emotional discomforts (3). According to the World Health Organization, depression is a leading cause of disability worldwide, with serious implications for educational and work performance (4, 5). Graduate students are particularly vulnerable to depression (6, 7), with poor mental health becoming increasingly common within this group. Research indicates that approximately 34% of graduate students experience depressive symptoms (8), making them six times more likely to experience depression compared with the general population (9, 10). The increasing prevalence of depression among graduate students highlights the urgent need to address this issue. Stressors contributing to mental health issues among graduate students include academic pressure, financial concerns, career planning, and family issues (11–13). Furthermore, graduate students may face academic bullying or violence from peers or mentors (14). Thus, the alleviation of depression among graduate students has become a pressing concern. However, although several studies indicate that graduate students are more prone to mental health symptoms than the general population, more research is still needed to provide further clarity. This study focused on graduate students to offer insights into and considerations for alleviating their depressive symptoms.

### 1.1 Relationship between neuroticism and depressive symptoms

Personality is a significant factor in the development of mental health issues, with neuroticism being a key trait that reflects individual tendencies towards negative thoughts and emotions (15). Neuroticism is characterized by a heightened response to stressors, frequent negative emotions, and maladaptive behavior (16, 17). Individuals with high levels of neuroticism often experience unstable emotions and intense reactions, particularly in response to negative events (18). Furthermore, high levels of neuroticism are linked to biases in information processing and a greater likelihood of experiencing emotional difficulties (19).

A study conducted with a cohort of first-year graduate students in China revealed that high levels of neuroticism significantly predict depression onset (20). The connection between neuroticism and depression can be explained through the vulnerability model, which shows that neuroticism is associated with high levels of negative emotions and low levels of positive emotions, which contribute to the development of depression (21). Furthermore, chronic negative emotional traits linked to neuroticism may result in maladaptive responses to stressors, increasing the likelihood of experiencing despair and ultimately developing depression (22). Although existing research findings suggest that neuroticism plays a role in the etiology and maintenance of depression (23, 24), little is known regarding how these associations influence the mental health of graduate students, and the factors underlying this relationship have not been sufficiently explored. Therefore, this study posits a significant positive correlation between neuroticism and depressive symptoms (H1).

### 1.2 Potential mediating effect of social support

Previous research has indicated that social support is a significant predictor of mental health (25, 26). Social support can be defined as the exchange of material and spiritual resources between individuals, leading to feelings of respect, care, and the ability to receive help (27, 28). Thus, social support is a feasible candidate for mediating the relationship between neuroticism and depression (29). Graduate students with sufficient social support may receive more assistance from family, friends, or classmates, thus helping to provide them with the resources and capabilities necessary to manage negative emotions when affected by adverse events.

A negative correlation has been established between social support and depression (30). For instance, during the COVID-19 pandemic, graduate students who were unable to engage in direct social interactions with mentors and peers exhibited relatively high levels of depression and social anxiety (31). Consequently, heightened social support not only protects graduate students against the progression of depression (32) but also enhances their happiness (33). These findings are consistent with the analysis of social psychological theories (34).

In contrast, neuroticism is negatively associated with social support (35). Higher levels of neuroticism are often linked to negative emotional states that can harm interpersonal relationships, thereby affecting opportunities to receive social support (36). Research also indicates that higher levels of neuroticism decrease perceived social support and contribute to depressive symptoms (37). Consequently, it can be inferred that the influence of neuroticism on depression symptoms among graduate students is mediated by social support (H2).

### 1.3 Potential mediating role of positive coping

In addition to social support, coping strategies play a crucial role in regulating the relationship between individual characteristics and adaptive outcomes (38). Coping styles reflect the specific behaviors and psychological efforts that individuals use to manage, endure, reduce, or minimize the impact of stressful events (39). Positive coping strategies, such as planning, seeking advice, and engaging in activities, can help mitigate the impact of stressors and mitigate or prevent emotional distress (40, 41). Some studies have indicated that graduate students who adopt positive coping strategies exhibit high levels of psychological resilience and lower levels of depression, anxiety, and stress (42, 43). Individuals who use positive coping strategies typically possess a fighting spirit and demonstrate better emotional expression, which is thought to signify enhanced psychological adaptability (44, 45). Conversely, negative coping mechanisms such as smoking and excessive drinking have been associated with high levels of depressive symptoms among graduate students (46). Furthermore, neurotic traits are closely related to coping ability. Thus, individuals with high and persistent levels of neuroticism are less likely to adopt positive coping strategies (47).

The stress coping model posits that stress can elicit psychophysiological fragility, such as neuroticism, perceptual control, and sensitivity, resulting in elevated resting arousal levels and irritability. Individuals equipped with effective coping mechanisms to manage heightened arousal can proactively navigate stressors and safeguard their mental well-being (48, 49). Previous studies indicated that coping strategies mediate the relationship between medical graduate students’ perceived stress and anxiety/depression (50). Positive coping mechanisms help reduce the impact of perceived stress on depression and anxiety among graduate students, whereas negative coping strategies exacerbate the influence of stress on depression and anxiety. Furthermore, coping mechanisms may play a mediating role in the relationship between neuroticism and mental disorders, with neuroticism associated with lower levels of positive coping and higher rates of mental health symptoms (51). Therefore, we hypothesize that the relationship between neuroticism and depressive symptoms in graduate students is mediated by the presence of positive coping strategies, drawing on both theoretical frameworks and empirical findings (H3).

### 1.4 Chain mediating effect of social support and positive coping

As hypothesized, both social support and positive coping strategies may mediate the relationship between neuroticism and depressive symptoms. Social support and coping strategies are closely intertwined (52) and recognized as factors that can bolster one’s capacity to overcome mental health challenges. Apart from the individual mediating roles mentioned above, a chain-mediating mechanism may exist that influences the link between neuroticism and depressive symptoms. The relationship between social support and positive coping, when both are considered mediating factors of neuroticism in depressive symptoms, remains unknown, including which plays a more significant mediating role. Previous studies have found that symptoms of depression and anxiety are significantly correlated with family functioning, social support, and coping strategies (53) as well as the mediating effect of coping strategies on social support and individual psychological distress (54). Individuals with adequate social support tend to adopt positive coping strategies based on the belief that their social networks offer substantial support and their coping endeavors are effective (55). Individuals with lower levels of neuroticism are more likely to receive heightened social support and engage in positive coping strategies, which are inversely related to depression and anxiety (38). Hence, it is plausible to infer that social support and positive coping may act as sequential mediators between neuroticism and depressive symptoms in graduate students (H4).

The four hypotheses are as follows: (H1) Neuroticism positively predicts depression symptoms among graduate students; (H2) neuroticism indirectly predicts depressive symptoms among graduate students through the mediating role of social support; (H3) neuroticism indirectly predicts depressive symptoms among graduate students through the mediating role of positive coping; and (H4) neuroticism may indirectly predict depression symptoms in graduate students through the chain mediated effect of social support and positive coping mechanisms.

## 2 Materials and methods

### 2.1 Participants

A cross-sectional survey of graduate students at a university in Dalian, Liaoning Province, China was conducted between October 7 and 20, 2021. Survey data were collected using the Wenjuanxing Professional Survey website (www.sojump.com). This study was reviewed and approved by the Ethics Committee of Liaoning Normal University (LL2021045). After reading the informed consent form, participants could choose whether to move forward with this study and could withdraw at any time. The researchers ensured that they did not disclose any content or personal information regarding this study. A total of 1845 graduate students participated in our study, including 1475 female and 370 male participants. Their ages ranged from 20 to 41 years (M = 24.08; SD = 1.946). All study participants were graduate students.

### 2.2 Eysenck personality questionnaire

The Eysenck Personality Questionnaire is a self-report inventory developed by British psychologist Eysenck. The Chinese version of the EPQ has been revised (56). It is widely used in large-scale mental health surveys because of its ability to identify levels of neuroticism in specific populations. Recent studies have used the EPQ to measure neuroticism levels in both adolescent and graduate students (20, 57). The EPQ Neuroticism Subscale was used to evaluate individual personality traits. The Chinese adaptation of the EPQ-N was revised in 1983, yielding a Cronbach’s α coefficient of 0.771 (58). Neurotic personality is an emotional trait characterized by the tendency to quickly arouse and slowly dampen emotions when stimulated. The scale comprises 24 items, with participants asked to respond with either yes (1) or no (0). For example, one item is “ Do you experience fluctuations in your mood?” The total score ranges from 0 to 24, with higher scores indicating higher levels of neuroticism. In the current study, Cronbach’s α coefficient for neuroticism was 0.67.

### 2.3 Beck depression inventory-II

The Beck Depression Inventory Second Edition (BDI-II) was used to assess depressive symptoms (59). It is the most widely used tool for measuring levels of depression and is used both as a screening tool and to assess the severity of depression in patients. In recent studies, the BDI-II has been used to assess depression in adolescents and college students (23, 60). There are a total of 21 items, ranging from 0 points (I don’t feel sad) to 3 points (I am so sad and in pain that I can’t bear it). For example, one of the items is “What is your attitude towards the future?” The total possible scores range from 0 to 63. Four levels of depression severity were determined based on the total score: no depression or mild depression (0-13), mild depression (14-19), moderate depression (20-28), and severe depression (29 and above). The Cronbach’s α coefficient of the Chinese version of BDI-II is 0.94 (61). The reliability analysis in this study indicated that the Cronbach’s α coefficient of the scale was 0.88.

### 2.4 Social support rating scale

We measured social support using the Social Support Rating Scale (SSRS) (62). Since 1990, the SSRS has been widely used to assess social support among different groups in China. In recent studies, the SSRS has been used to assess social support among pregnant women and medical graduate students (29, 33). This 10-item scale includes three dimensions: objective support, subjective support, and utilization of support. For example, one item is “How many close friends do you have who can provide you with support and help?” The SSRS typically uses multi-axis evaluation methods. A total score below 20 indicates a low level of social support, 20–30 indicates average social support, and 30–40 indicates satisfactory social support.

A 1994 study on reliability and validity showed that the retest reliability after two months was 0.92, and the inter-project agreement was 0.89 ~ 0.94 (63). In this study, the reliability analysis revealed a Cronbach’s α coefficient of 0.848 for this scale.

### 2.5 Simplified coping style questionnaire

The Simple Coping Style Questionnaire (SCSQ) was developed and revised by Xie and is applicable to the Chinese population (64). The SCSQ is designed to assess the methods and strategies individuals use to cope with stress and adversity. Its purpose is to explore the common coping mechanisms that individuals use when faced with stress and challenges and their effectiveness. The SCSQ has been used in recent studies to evaluate coping strategies among college and graduate students (43, 65). It consists of 20 items rated on a 4-point scale (0 = non-acceptance, 3 = frequent acceptance) and measures two dimensions: positive and negative coping. Positive coping styles include Items 1–12. Example questionnaire items are “I strive to see the positive side of things” and “release myself through work, study, or other activities.” In a previous study, Cronbach’s α coefficient for this scale was 0.90, with positive and negative coping strategies showing alpha coefficients of 0.89 and 0.78, respectively (64). The SCSQ has shown good reliability among Chinese college students (66). In this study, the Cronbach’s α coefficient for positive coping was 0.80.

### 2.6 Statistical analysis

The analysis in this study was conducted using SPSS software (version 26.0). SPSS is a sophisticated statistical analysis program that is highly user-friendly, flexible, and scalable. Therefore, it is an ideal choice for professionals engaged in questionnaire surveys and analyses. In recent studies, SPSS has been used to conduct mediation analyses among various populations (32, 37, 38). We first compared the descriptive statistics and demographic variables (age, gender) and four scale scores (neuroticism, depressive symptoms, social support, and positive coping). Pearson’s correlation analysis was used to explore the relationships among neuroticism, social support, positive coping, and depressive symptoms. Skewness and kurtosis tests were used to comprehensively test the normality of the distribution of scores on the four scales. The SPSS PROCESS macro 4.0 was used to investigate the mediating role of social support and positive coping between neuroticism and depressive symptoms (67). The mediating effect of social support and positive coping was tested by applying PROCESS Model 6. A bootstrap procedure with bias correction was used to calculate indirect effects. If the 95% confidence interval (CI) did not include zero, the mediating effect was significant (68). Gender and age were included as covariates in this model.

## 3 Results

### 3.1 Common method variance testing

Harman’s univariate test was conducted to examine the common method bias caused by self-reported scales (69). The first factor accounted for 10.37% of the total variation, which is lower than the 40% value (70). This indicates that common method biases are unlikely to confuse the interpretation of data analysis results (68).

### 3.2 Descriptive analysis and correlation between variables

Basic descriptive data on neuroticism, social support, positive coping, and depressive symptoms are shown in **Table 1**. Specifically, the average total scores were 10.64 ± 3.38 for neuroticism, 25.83 ± 5.80 for social support, 30.56 ± 8.96 for active coping, and 6.24 ± 6.80 for depressive symptoms. The average score for gender is 1.20 ± 0.04, and the average score for age is 24.08 ± 1.94.

**Table 1:** Means and standard deviations of the research variables .

Variables	Mean	SD
1. Neuroticism	10.64	3.38
2. Social support	30.56	8.96
3. Positive coping	25.83	5.80
4. Depressive symptoms	6.24	6.80
5. Gender ^a^	1.20	0.40
6. Age	24.08	1.94

N = 1845.

a Gender was treated as a dummy variable: 1 = female, 2 = male.

We conducted skewness and kurtosis tests to comprehensively examine the distribution normality of the aforementioned variables. A study published in 2013 showed that when the sample size is greater than 300, an approximately normal distribution is defined for variables with skewness less than 3 and kurtosis less than 8 (71). As shown in **Table 1**, except for age, the skewness values of each variable range from -0.39 to 1.91, and the kurtosis values range from -0.28 to 6.58, indicating a normal distribution. Pearson’s correlation analysis was used to test the correlation between the variables. As shown in **Table 2**, all the variables were significantly correlated. Neuroticism was negatively correlated with social support (*r* = -0.24, *p* < 0.01) and positive coping (*r* = -0.19, *p* < 0.01) and positively correlated with depressive symptoms (*r* = 0.50, *p* < 0.01). Depressive symptoms were negatively correlated with social support (*r* = -0.41, *p* < 0.01) and positive coping (*r* = -0.34, *p* < 0.01). Social support was positively correlated with positive coping (*r* = 0.40, *p* < 0.01). Gender was positively correlated with neuroticism (*r* = 0.07, *p* < 0.01) and depressive symptoms (*r* = 0.09, *p* < 0.01) and negatively correlated with social support (*r* = -0.06, *p* < 0.01). Age was positively correlated with gender (*r* = 0.13, *p* < 0.01).

**Table 2:** Skewness, kurtosis, and correlations of the research variables.

Variables	Skewness	Kurtosis	1	2	3	4	5	
1. Neuroticism	0.44	0.14	1					
2. Social support	0.01	-0.28	-0.24**	1				
3. Positive coping	-0.39	-0.17	-0.19**	0.40**	1			
4. Depressive symptoms	1.91	6.58	0.50**	-0.41**	-0.34**	1		
5. Gender	1.49	0.24	0.07**	-0.06**	-0.03	0.09**	1	
6. Age	3.24	17.02	0.01	-0.02	0.01	0.04	0.13**	1

N = 1845.

All tests are two-tailed. This table presents the general means, standard deviations, skewness, kurtosis, and correlations among the four major variables. ** indicates a significant correlation between the variables, p < 0.01.

### 3.3 Social support and positive response: chain mediation effect analysis

A significant correlation was observed among neuroticism, depressive symptoms, social support, and positive coping, meeting the statistical requirements for further mediating effect analysis between neuroticism and depressive symptoms (72). We analyzed the mediating role of social support and positive coping in the relationship between neuroticism and depressive symptoms using Model 6 of the PROCESS macro in SPSS 26.0, as developed by Hayes (67). The regression analysis results are presented in **Table 3**.

**Table 3:** Regression analyses of relationships between variables in the mediation model.

Dependent variable	Independent variable	*β*	*SE*	*t*	*R^2^*	*F*
Social support	Gender	-0.969	0.510	-1.899	0.064	42.214***
	Age	-0.095	0.105	-0.904		
	Neuroticism	-0.649	0.060	-10.854***		
Positive coping	Gender	-0.146	0.312	-0.469	0.171	94.576***
	Age	0.064	0.064	1.002		
	Neuroticism	-0.175	0.038	-4.662***		
	Social support	0.243	0.014	17.067***		
Depressive symptoms	Gender	0.633	0.319	1.983*	0.366	212.083***
	Age	0.101	0.066	1.548		
	Neuroticism	0.814	0.039	20.974***		
	Social support	-0.180	0.016	-11.454***		
	Positive coping	-0.204	0.024	-8.539***		

N = 1845.

This table presents the results of the multiple hierarchical regression analysis of neuroticism, social support, positive coping, and depressive symptoms. *p < 0.05, ***p < 0.001.

**Figure 1:**
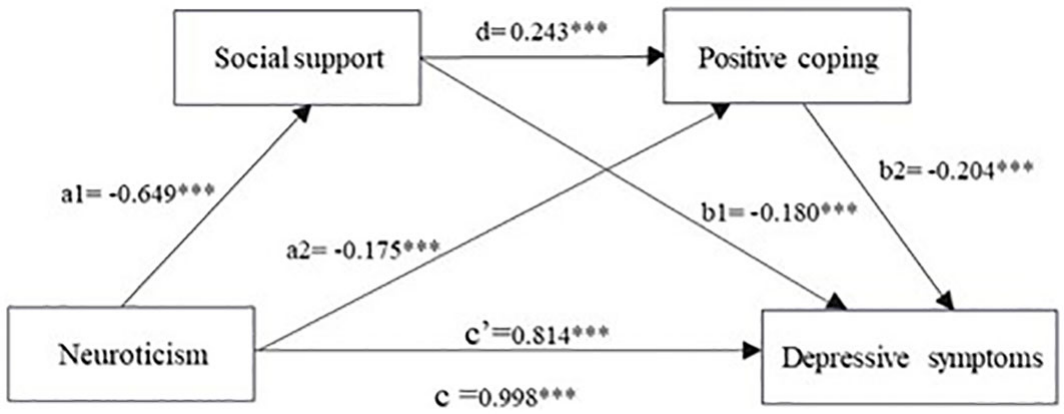
Chain mediation model of neuroticism, social support, positive coping, and depressive symptoms. ***p < 0.001.

Neuroticism and social support after controlling for gender and age (β = - 0.649, *p <* 0.001) and proactive response (β = - 0.175, *p* < 0.001) is negatively correlated with depressive symptoms (β = 0.814, *p <* 0.001) shows a positive correlation. Social support could positively predict positive responses (β = 0.243, *p* < 0.001) and negatively predict depressive symptoms (β = - 0.180, *p* < 0.001). In addition, positive coping was found to be an important negative predictor of depressive symptoms (β = - 0.204, *p* < 0.001). **Figure 1** shows the model diagram after testing.

**Table 4** shows the mediating roles of social support and positive coping in the correlation between neuroticism and depressive symptoms. **Figure 1** illustrates a chain-mediated model of neuroticism and depressive symptoms. Both **Table 4** and **Figure 1** indicate that social support and positive coping play important mediating roles between neuroticism and depressive symptoms. The total effect value of neuroticism on depressive symptoms was 0.998, the direct impact of neuroticism on depressive symptoms was 0.814, and the total mediating effect value was 0.185. The proportion of the mediating effect to the total effect was 18.53%. These mediating effects involve three pathways. Path 1: Neuroticism → Social Support → Depression Symptoms (0.117), Path 2: Neuroticism → Positive coping → Depression symptoms (0.036), and Path 3: Neuroticism → Social Support → Positive Coping → Depression Symptoms (0.032). The indirect effects of Paths 1– 3 accounted for 11.72%, 3.60%, and 3.21%, respectively. The 95% confidence interval for these indirect effects did not include zero; thus, all three indirect effects were significant. A comparison shows that the bootstrap 95% confidence interval for the differences between indirect effects for Paths 1 and 2, as well as between indirect effects for Paths 1 and 3, did not include zero, indicating a significant difference between Path 1 and Paths 2 and 3. When using the same comparison method between Paths 2 and 3, the bootstrap 95% confidence interval for the difference did include zero, indicating that no significant difference between them. These results indicate that neuroticism can not only predict depressive symptoms through a single mediating effect of social support and positive coping but also indirectly through a chain mediating effect of social support and positive coping.

**Table 4:** Social support and positive coping in the mediation analysis.

	Effect value	Boot SE	Boot LLCI	Boot ULCI	Effect proportion
Total effect	0.998	0.04	0.919	1.078	
Direct effect	0.814	0.039	0.738	0.890	81.56%
Total indirect effect	0.185	0.021	0.145	0.228	18.53%
Indirect effect 1	0.117	0.016	0.086	0.150	11.72%
Indirect effect 2	0.036	0.010	0.018	0.057	3.60%
Indirect effect 3	0.032	0.006	0.021	0.044	3.21%
Compare 1	0.081	0.020	0.042	0.120	
Compare 2	0.084	0.016	0.055	0.117	
Compare 3	0.004	0.010	-0.016	0.023	

N=1845.

The direct and indirect effects of neuroticism on depressive symptoms are shown in this table. Boot SE, Boot LLCI, and Boot ULCI refer to the standard error and lower and upper limits of the 95% confidence interval of the effects, respectively, estimated using the percentile bootstrap method with deviation correction. Indirect effect 1: neuroticism → social support → depressive symptoms; Indirect effect 2: neuroticism → positive coping→ depressive symptoms; Indirect effect 3: neuroticism → social support→ positive coping→ depressive symptoms. Compare 1: indirect effect 1-indirect effect 2; Compare 2: indirect effect 1-indirect effect 3; Compare 3: indirect effect 2-indirect effect 3.

## 4 Discussion

This study established a chain-mediation model to explore the relationship between neuroticism and depressive symptoms among graduate students. We have confirmed a positive correlation between neuroticism and depressive symptoms, partially mediated by social support and positive coping through three pathways: social support, positive coping, and social support → positive coping. These studies will help us gain a deeper understanding of the relationship between neuroticism and depressive symptoms among graduate students and provide guidance for effectively reducing neuroticism levels and alleviating depressive symptoms.

### 4.1 Effect of neuroticism on depressive symptoms in graduate students

Characteristics such as emotional instability, irritability, anxiety, and impulsivity are indicative of stress and depression onset, which increases the risk of depression (23, 73). Several studies have demonstrated a positive association between higher levels of neuroticism and depressive symptoms, both under normal circumstances and during the COVID-19 pandemic (74–77). Neuroticism is recognized as a specific risk factor or cognitive vulnerability for the development and persistence of depressive symptoms such as sadness, distress (78) and severe depression (79).

As the reserve talent of a scientific research team, graduate students face many unknown pressures and uncertain factors in the process of academic exploration and future employment. Previous research has underscored the predictive influence of neuroticism on depression among graduate students (20). Neuroticism involves heightened sensitivity to emotional stimuli and elicitation of physiological responses to stressors (80). Investigating the relationship between neuroticism and depressive symptoms could inform targeted intervention strategies. Understanding the impact of neuroticism and its correlation with depressive symptoms is of practical significance. Lowering neuroticism levels in graduate students and fostering a healthy personality not only benefits their mental well-being but also influences their future career trajectories. It is crucial to monitor graduate students’ neuroticism and implement tailored coping mechanisms to alleviate their depressive symptoms.

### 4.2 Mediating effect of social support

Our findings demonstrate that social support mediates the association between neuroticism and depressive symptoms, which is in line with previous research (29, 81). Individuals with higher neuroticism scores may experience reduced social support and are less likely to seek or provide support, thereby increasing their risk of depression (29). Increased social support protects against depression onset in graduate students (82) and fosters a heightened sense of connectedness, which is consistent with psychosocial theory (34). Furthermore, the study by McHugh and Lawlor (81) unveiled the mediating function of social support from neighbors in the relationship between neuroticism and heightened depression levels. Research has also indicated that social support and life events are dynamic elements that modify the longitudinal progression of the link between neuroticism and depressive symptoms in college students, leading to cumulative effects over time (30).

Neuroticism has been widely reported as a risk factor for depression (21, 23). According to our findings, neuroticism can directly influence graduate students’ depressive symptoms and indirectly affect them through social support. Some studies have also indicated that neuroticism can affect the provision and acceptance of social support, particularly social support, thereby reducing perceived support (29, 83). This indicates that, if individuals respond to high levels of social support, the positive correlation between neuroticism and depressive symptoms weakens. Therefore, sufficient attention should be paid to improving the social support system for graduate students to reduce their emotional distress and the risk of mental illness.

### 4.3 Mediating effect of positive coping

In line with previous research (38, 57), this study reaffirms that positive coping serves as a mediator in the correlation between neuroticism and depressive symptoms. Employing positive coping strategies elicits positive emotions and behaviors, consequently enhancing psychological well-being. Embracing positive coping mechanisms can assist graduate students in nurturing sound psychological states and effectively managing stressful life events. A recent study indicated that individuals exhibiting low psychological flexibility and high neuroticism show greater amelioration of depressive symptoms upon integrating positive coping strategies (84). Further research has shown that programs that increase positive emotions, adaptive coping strategies, and resilience can reduce the likelihood of anxiety or depression symptoms in graduate and postdoctoral populations (43, 85). Our results underscore the inverse relationship between positive coping and depressive symptoms, suggesting that graduate students with lower levels of neuroticism tend to embrace positive coping strategies and encounter fewer depressive symptoms. Hence, clinical interventions should prioritize the augmentation of positive coping strategies to aid graduate students with heightened neuroticism in adapting to stressful life events, thereby mitigating the prevalence of depression and anxiety.

### 4.4 Chain mediating role of social support and positive coping

In addition to the two mediating effects mentioned above, we found that social support and positive coping had a chain-mediating effect on the relationship between neuroticism and depressive symptoms. Thus, neuroticism may weaken social support networks, leading to a reduction in adaptive coping mechanisms and ultimately exacerbating an individual’s depressive symptoms. Social support and coping strategies exhibit strong interdependence (86), with higher levels of social support enhancing college students’ emotional well-being. The effective utilization of social support facilitates the adoption of positive coping strategies. Both social support and positive coping strategies served as crucial mediating variables in the interplay between personality traits and emotional states. Previous research has demonstrated the mediating role of social support and positive coping in the relationship between personality traits and symptoms of depression and anxiety (38). Studies on adolescent depression interventions underscore the significance of personality traits, family environment, coping styles, and other factors (87, 88). Our current investigation builds upon these findings, specifically examining the impact of neuroticism on depressive symptoms in graduate students, and highlights the chain mediating role of social support and positive coping. Our findings underscore the importance of healthy personality traits, supportive social networks, and effective coping strategies in preventing and alleviating depressive symptoms among graduate students.

### 4.5 Limitations and implications

This study investigated the relationship between neuroticism and depressive symptoms in graduate students. Our findings suggest that neuroticism influences depressive symptoms through three pathways: the direct impact of social support, the direct impact of positive coping, and the combined impact of social support and positive coping. However, the limitations of this study are important to acknowledge. First, it employed a cross-sectional design, and caution should be exercised when inferring causal relationships from the findings. Subsequent research should consider longitudinal studies to establish a causal relationship between neuroticism and depressive symptoms in graduate students. Second, most of the sample in this study consisted of female graduate students; thus, the generalizability of the results to a wider student population may be limited. Furthermore, this study did not examine the potential influence of certain external or underlying mediating variables. The characteristics of graduate student participants, such as differences in their majors or the severity of neuroticism, may have affected their depressive symptoms.

Despite these limitations, the results have important theoretical and practical implications. In terms of theoretical significance, analyzing the relationship between graduate students’ neuroticism, depressive symptoms, social support, and positive coping strategies can help clarify the mediating mechanism of neuroticism on depressive symptoms. In terms of behavioral interventions, emotional and social skills training benefits postgraduate students by increasing positive emotions and psychological resilience, thereby improving their social support networks of postgraduate students. Additionally, intervention measures should focus on helping graduate students utilize problem-focused coping strategies (e.g., seeking information to address stressors) to counteract negative psychological issues and mental disorders.

## 5 Conclusion

Understanding the correlation between neuroticism and depressive symptoms in graduate students is imperative to improve their mental health and long-term progress. This study examined the impact of neuroticism on depressive symptoms among recently admitted graduate students, as well as the mediating roles of social support and positive coping strategies. This study’s outcomes indicate that bolstering social support for these students may enhance their tendencies towards employing positive coping mechanisms, thereby aiding in the alleviation of depressive symptoms.

## Data Availability

The raw data supporting the conclusions of this article will be made available by the authors without undue reservation.
